# Passive acoustic monitoring of sperm whales and anthropogenic noise using stereophonic recordings in the Mediterranean Sea, North West Pelagos Sanctuary

**DOI:** 10.1038/s41598-022-05917-1

**Published:** 2022-02-07

**Authors:** Marion Poupard, Maxence Ferrari, Paul Best, Hervé Glotin

**Affiliations:** 1 Université de Toulon, Aix Marseille Univ., CNRS, LIS, DYNI, Marseille, France; 2 Université de Toulon, INPS, SMIoT, Marseille, France

**Keywords:** Data acquisition, Behavioural ecology

## Abstract

A total of 147 days spread over 4 years were recorded by a stereophonic sonobuoy set up in the Mediterranean sea, near the coast of Toulon, south of France. These recordings were analyzed in the scope of studying sperm whales (*Physeter macrocephalus*) and the impact anthropic noises may have on this species. With the use of a novel approach, which combines the use of a stereophonic antenna with a neural network, 226 sperm whales’ passages have been automatically detected in an effective range of 32 km. This dataset was then used to analyze the sperm whales’ abundance, the background noise, the influence of the background noise on the acoustic presence, and the animals’ size. The results show that sperm whales are present all year round in groups of 1–9 individuals, especially during the daytime. The estimated density is 1.69 whales/1000 km$^2$. Animals were also less frequent during periods with an increased background noise due to ferries. The animal size distribution revealed the recorded sperm whales were distributed in length from about 7 to 15.5 m, and lonely whales are larger, while groups of two are composed of juvenile and mid-sized animals.

## Introduction

The sperm whale (*Physeter macrocephalus*) is a cosmopolitan species found all around the globe. The Mediterranean population is considered to comprise less than 2500 mature individuals^1^ and is listed as Endangered in the Red List of Threatened Species of IUCN^2^. Like the eight common cetacean species inhabiting the Northwestern Mediterranean Sea, sperm whales evolve in a highly anthropized environment^3^. Sharing an environment with a dense human activity implies threats for the animals such as bycatch^4,5^, vessel collisions^6^, or ingestion of solid debris^7^. Besides the latter, one of the main anthropic pressure in the marine environment is acoustic^8^. The dense marine traffic and military activities induce noises that, for other cetaceans, have been shown to trigger behavioral changes, acoustic masking, and hearing loss^9^. It seems therefore relevant to conduct scientific studies to increase the knowledge about sperm whales and the impact of marine traffic on their habitat use.

Scientists use many different methods to study cetaceans in the wild, such as photo-identification^10^, genetic sampling^11^, mark-recapture or acoustic recordings. Visual approaches are to this date the only ways to identify individuals, to state on their body conditions, and to measure group sizes reliably, which makes them essential. Nonetheless, these approaches demand costly sea expeditions, especially challenging due to the fact that sperm whales spend only 10% of their time at the surface^12^. Oleson and colleagues^13^ support this idea, showing that in a comparative study, visual observers did not detect any sperm whales when acoustic observers did. Overall, the two approaches are complementary to study sperm whale populations^14,15^, and visual data could validate acoustic estimations.

Several acoustic approaches exist to monitor cetaceans. One of them is to attach acoustic tags on their body^16^, which might alter their behavior leading to an observation bias^17,18^. Another approach is to tow hydrophones behind a monitoring vessel^19^, which requires high human effort and yields relatively noisy recordings. Passive Acoustic Monitoring (PAM) using autonomous recorders avoids those challenges, is particularly suited for long-term surveys, and thus is widely used in underwater bioacoustics^20,21^. PAM can be used to answer several scientific questions such as population density estimation^21^, acoustic presence and characterization^22^, as well as behavior in hardly accessible environments such as deep waters.

During dives, sperm whales emit trains of clicks, whereas, for socialization, they emit small rhythmic series of clicks (Codas). Sperm whales have the most powerful bio-sonar in the animal kingdom (the loudest recorded click was at 230 dB re: 1 $\upmu$Pa rms^23^). In 1972, a first study correlated the impulsive sound from sperm whales and the morphology of the animal^24^. They showed that the animal creates an initial pulse at the front of its head, in the ‘museau de singe’ (aka. monkey lips), which will then bounce back and forth in its head, passing through multiple oil sacs, before exiting. The initial sound will leak forward into the water (forming the pulse P0) and also propagate backward within the animal’s head (passing through the spermaceti), before being reflected forward (by the air-filled nasofrontal sac): it is the Pulse 1 (P1). This reflection happens several times, forming the P2, P3 etc. In 1972, Norris et al.^24^ proposed that the Inter-pulse Interval (IPI) describes the time sound takes to travel the head of the sperm whale and could be linked to its size (the length of the head being correlated to the total whale size^25^). Following this, several studies confirmed this hypothesis between the animal length and the IPI^23,26–28^.

In this study, we compute the IPI of detected clicks in order to estimate the size distribution of sperm whales in the area. In the past, different approaches have been used to estimate the IPI^29^, such as displaying the waveform of the signal and the spectrogram^30–32^, the cross-correlation of the signal waveform (giving the time delay between the pulses in a click)^33^, and the analysis of the cepstrum^32,34^. While some studies have compared the manual and automatic methods to find IPI^29,32^, we propose a novel tool to offer annotators different types of visualizations (waveforms, spectrograms, cepstrums, and cross-correlations) to help reduce annotation errors.

The objective of this research is to set up an acoustic protocol and relevant methods to determine the number of individuals in an area, to estimate their size, density, and the influence of noise on their attendance. The latter took form as the BOMBYX sonobuoy, installed in 2015 at a 25 m depth and with two hydrophones. A large number of recordings were yielded, such that automated detection methods were required. The analysis following click detection included Time Delays of Arrival (TDoAs), IPI, background noise level computations, and the estimation of the sperm whale density. They revealed some insights into how sperm whales evolve in the area, and how they react to anthropogenic noises.

## Results

The results of this study are presented in 5 main parts: the sperm whales’ acoustic presence, the Background Noise (BN), the influence of BN on the Acoustic Presence (AP), the animals’ size distribution, and the animal density.

### Sperm whale acoustic presence

**Figure 1:**
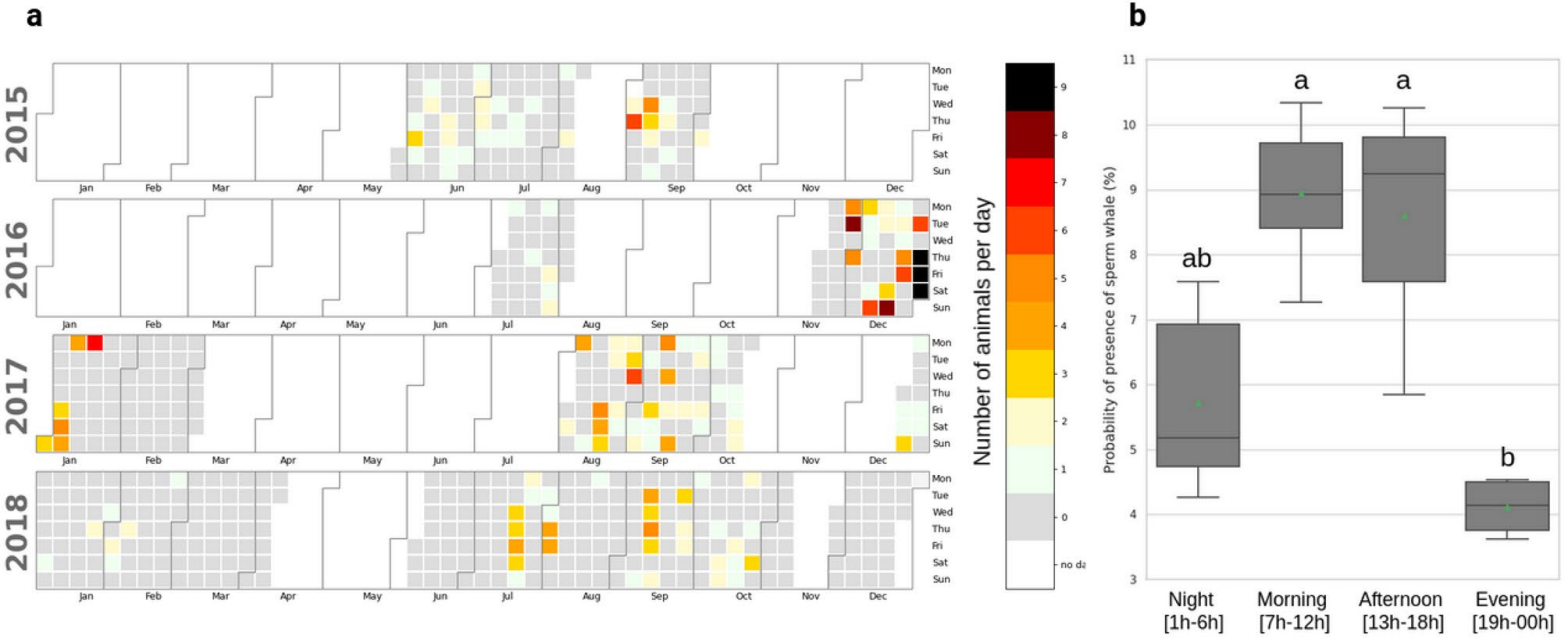
Left (**a**): the Number of detected sperm whales per day during the 4 years of recordings (white region: *no d = no data*). Right (**b**): mean probability of presence for each period of the day.

The analysis of the 3532 recorded hours (from 2015-05-30 to 2018-12-26) revealed the occurrences of sperm whales throughout these 4 years. In total, 226 sperm whale passages have been recovered (total of 347 individuals). Figure 1 presents the number of detected individuals each day during the 4 years of recording, with white regions indicating no recordings. Sperm whales were found all year round, with no particular seasonal cycle. Some periods were more densely visited than others, for example, December 2016 and January 2017. The number of animals per passage varied from 1 to 9 individuals. The distribution of the duration of the passages is presented in Fig. S4 (Supplementary Material). The mean passage duration is 4 h, the median is 3 h, the maximum is 19 h (9 tracks at the same time), the minimum is 10 min.

To evaluate dial patterns of acoustic presence, the probability of presence over hours was computed. The maximum probability was found at noon (10.5%) and the minimum at 9 PM (3.7%). Averaging probabilities into four periods (Night, Morning, Afternoon, and Evening) shows a significant difference of the probability of presence throughout the day, Kruskal–Wallis test (*p value*= 0.001 $\leqslant \alpha$, H statistic = 16.7) (see Fig. 1). The Dunn–Bonferroni test showed a statistical difference (0.002 and 0.005 $\leqslant \alpha$) in the sperm whale probability of detection during the daytime with fewer sperm whale clicks occurring in the evening compared to the morning and afternoon (Fig. 1).

We were also able to extract the number of animals for each passage, counting the simultaneous TDoA tracks. More than half of the passages are made up of a single individual (121 passages), 50 passages are made up of 2 individuals, and 23 with 3 individuals. The maximum number of individuals in a passage is 9.

### Background noise analysis

**Figure 2:**
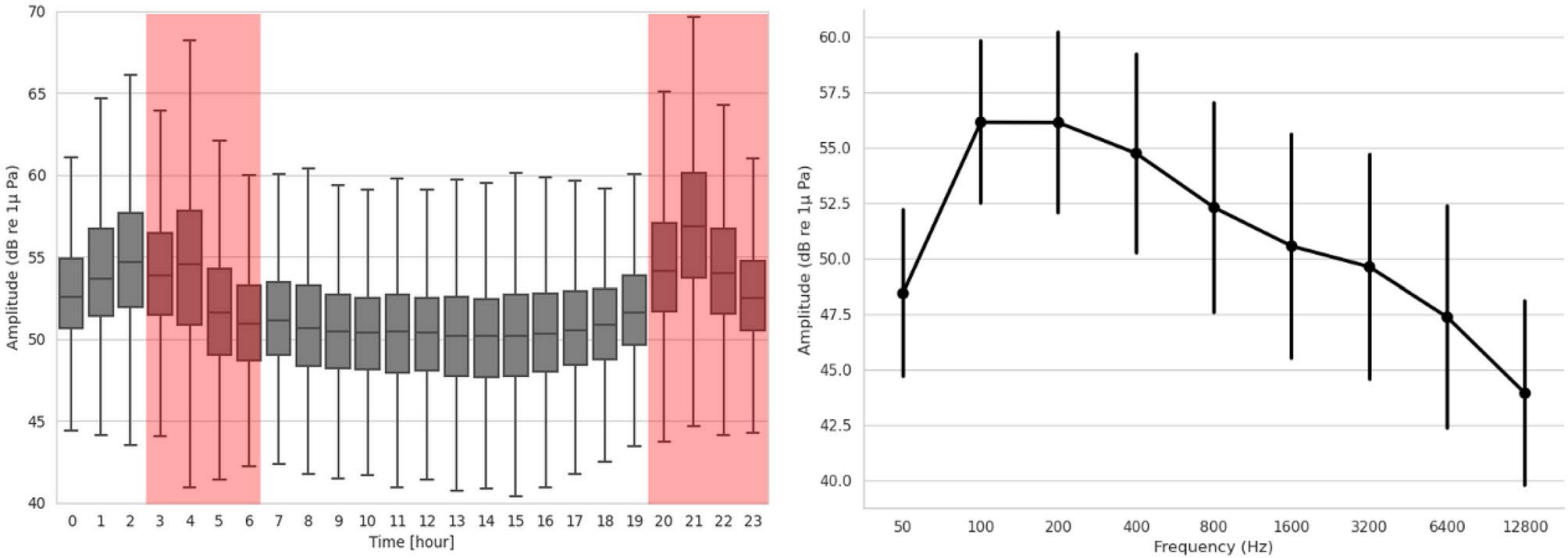
Left: daily pattern of the amplitude for the 800 Hz octave for all records. The red regions represent the ferry period. Right: average of the amplitude for each of the nine octaves, with vertical bars indicating the standard deviation.

To assess the performance of the detector a Convolutional Neural Network (CNN) as well as to measure the impact of noise on the presence of sperm whales, the amplitudes of different octave bands were computed and analyzed. The distribution of the background noise (octave 800 Hz) according to hours day is shown in Fig. 2 (left). All octaves’ dial distributions have the same shape as the octave 800 Hz, with the energy peaking around 4 AM and 9 PM. The study area is frequented daily by ferries, connecting Toulon or Marseille to Corsica as seen by their scheduled times between 3 AM and 6 AM and from 8 to 9 PM (see the red regions of Fig. 2). The closest ferry route is approximately 3km away from the antenna. The results showed a significant difference for all octaves between the amplitudes during the ferry and not ferry periods (Mann–Whitney test, *p value* < 0.05). The average of background noises increased by approximately 3 dB during the ferry periods, while the ferry crossings are only a few kilometers away from the antenna (Fig. 5). The baseline sound intensity for ferries (160 feet long, traveling at 23 knots) in Canada was measured between 183 and 192 dB^35^.

The right part of Fig. 2 shows the evolution of the amplitude for each octave. This result is consistent with the ambient noise spectra schematics by Wenz^36^. For the latter measurements, no statistical differences were found between months or seasons. The differences were not significant for all octaves (Kruskal–Wallis *p value*>0.05). The period of the year, therefore, does not influence the sound pressure levels. On the other hand, the results showed a significant difference between sound pressure levels during daytime versus night time, and so for all octaves (Shapiro–Wilk’s test *p value*= 0.001 < 0.05, Mann–Whitney test *p value*= 0.002 < 0.05). Noise levels were higher on average at night than during the daytime, for all octave bands. This increase in noise may be caused by the presence of ferries during these time slots (red part on the Fig. 2).

### Sperm whale acoustic detection and background noise

Anthropogenic noises negatively influence marine mammals by affecting their abundance^37^, their behavior^38^, and numerous processes of importance for their well being^39^ (orientation, reproduction, communication). This influence depends on many acoustic features including the intensity, the bandwidth, or the duration of the exposure. In this study, we compared the evolution of the sound pressure level according to the presence/absence of sperm whales.

**Figure 3:**
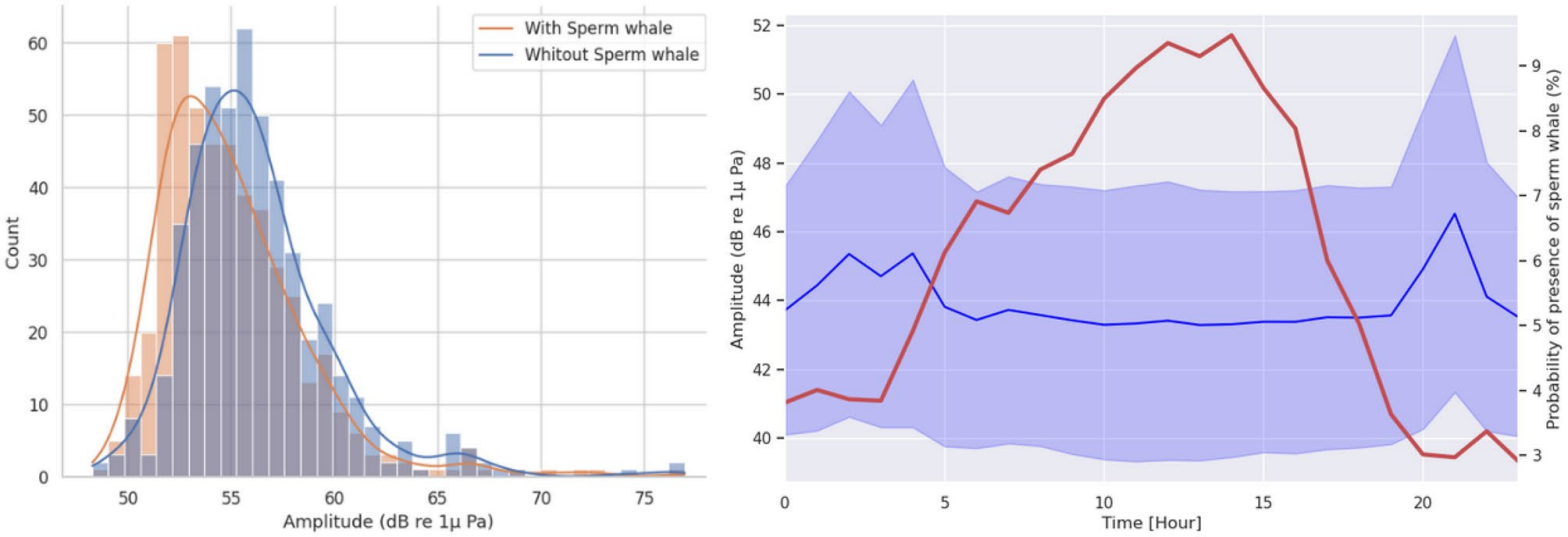
Left: Distribution of the amplitude for the octave 12,800 Hz according to presence/absence of sperm whales. Right: Superposition of dial pattern of amplitudes for the octave 12,800 Hz and probability of presence of sperm whales.

The results showed a significant difference between the amplitudes during the sperm whales’ presence/absence: Mann–Whitney U = 14.44, (sample size = 300), *p value* = 0.0008 < 0.05, for all octaves except 6400 Hz and 12,800 Hz (U = 122 and 145, (sample size =300), *p value* 0.182 and 0.230). Figure 3 shows the distributions of measured amplitudes for periods with and without sperm whales for the octave 12,800 Hz (this frequency was chosen since it lies approximately at the center of the acoustic emissions of the sperm whale). These results show that when sperm whales are present, the noise level is lower. Or in other words, sperm whales are statistically less present in noisier environments. This is further demonstrated in Fig. 3 right, where, during 4 AM and 9 PM (noise peaks), the presence of sperm whales is lowest.

**Figure 4:**
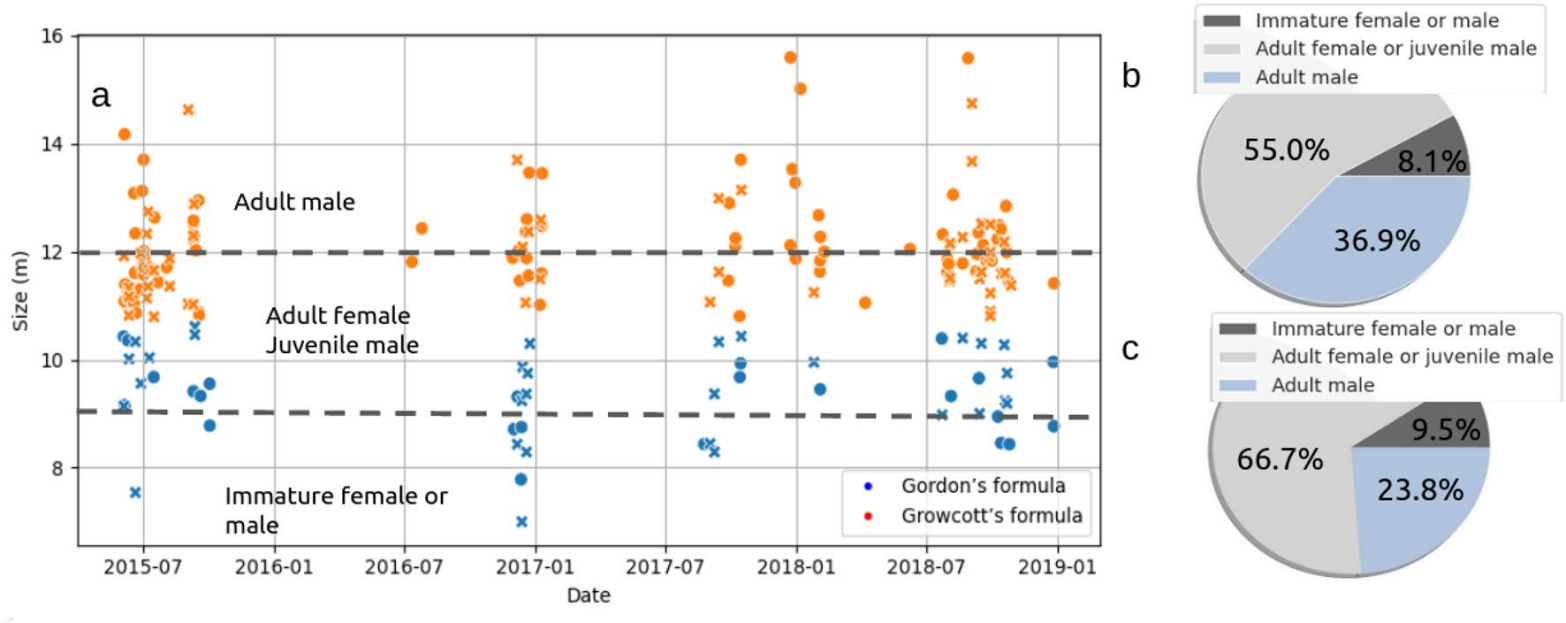
Left: size of the sperm whales over the 3 years of recordings for passages with one individual (dot) and two individuals (cross). Right: the proportion of each size categories for passage with one individual (**b**), and two individuals (**c**).

### Sperm whale interpulse interval (IPI) and size measurement

The data did not reveal any seasonal or yearly pattern concerning sperm whale size distribution (Fig. 4). During the 2018 sessions, we were able to record large individuals (probably adult males, over 15 m), not present in previous years. Furthermore, we see a greater proportion of adult males in passages with one individual than with those of two individuals (Fig. 4). Conversely, the proportion of juveniles is greater when there are two individuals in the group (9.5% vs 8.1%). This is consistent with the fact that adult males are solitary while females and young sperm whales stay in groups. Solitary passages thus significantly imply greater animal size compared to those with two animals (Mann–Whitney U = 3510, (sample size = 300), p value = 0.004).

The variability of sperm whale sizes for passages with a single individual was tested against the following other parameters with no significant statistical difference: **time** (month, year, season), **the sun** (sunrise, sunset), **the moon phase** (new moon, first quarter, full moon, last quarter).

### Sperm whale density

**Figure 5:**
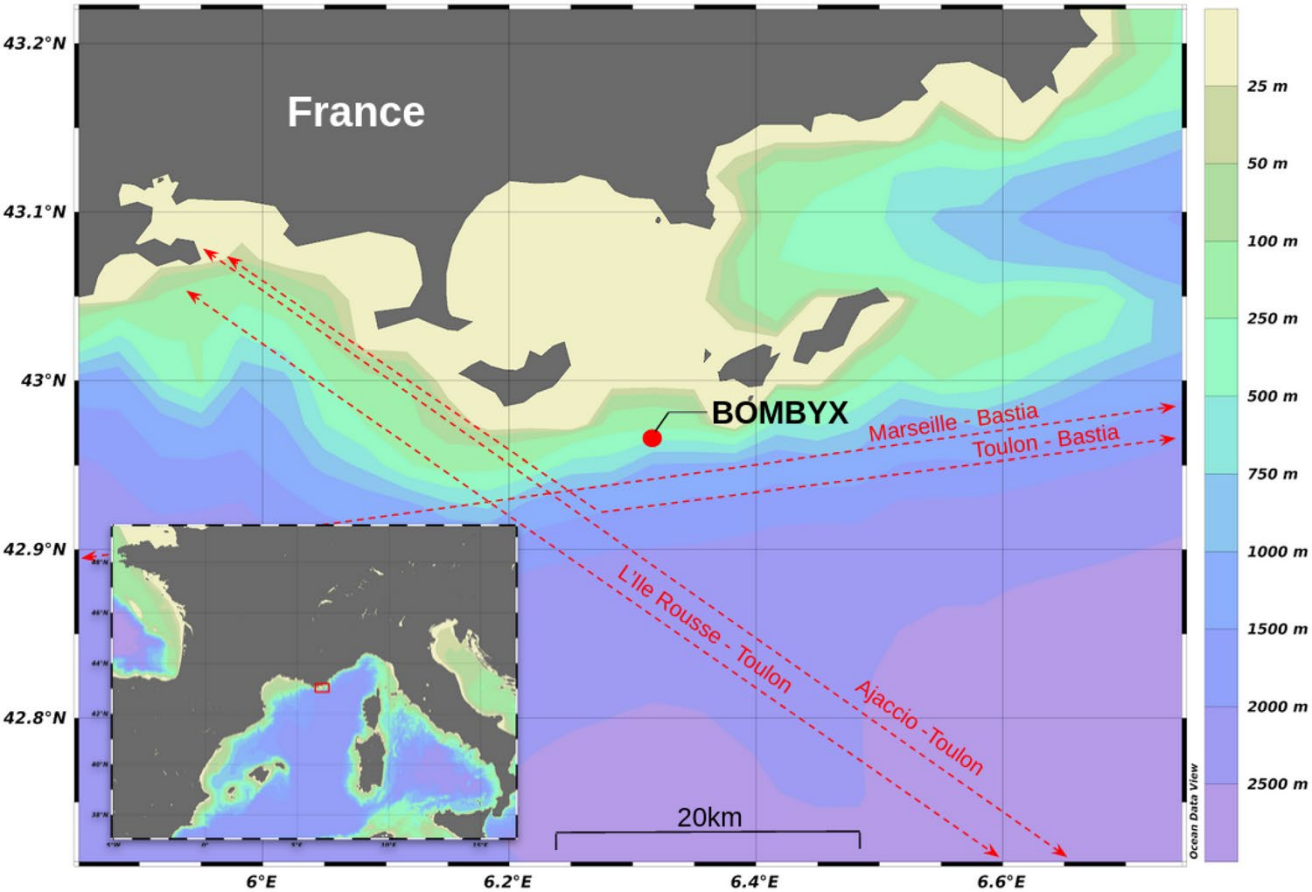
Up: Bathymetric map of the region showing the geographic location of the BOMBYX buoy and the ferry’s trajectories (red lines). The map was made with Ocean Data View (ODV) software^81^.

Figure 7 (right) gives the effective radius of the antenna depending on the background noise. For the average noise level of the 12,800 Hz octave (43.93 ± 4.17 dB re 1 $\upmu$Pa, see Fig. 2), this gives an effective radius of 32.9 ± 2.3 km. Since half of the covered area is shallow waters (Fig. 5), we assumed that only half of the area within this range could contain sperm whales^40^. This corresponds to an area of 1700 ± 237 km$^2$. With 422 sperm whales detected over a period of 147 days, the average density of sperm whales in the area was 1.69 ± 0.24 whales/1000 km$^2$.

## Discussion

The results obtained analyzing the 3532 h of recordings provided a first long-term survey about the presence of sperm whales on the French Mediterranean coast in the Pelagos sanctuary. The stereophony of the sonobuoy allowed us to compute TDoAs tracks, enabling an efficient browse of long-term data for annotation of presence/absence as well as for estimating the number of simultaneous individuals. The Mediterranean sperm whale subpopulation had already been studied at very large geographical scales^41–43^, while other populations were monitored over long time period such as Gordon et al.^44^ (4 months), Ward et al.^45^ (42 days), Ackleh et al.^46^ (4 month over 7 years), Caruso et al.^32^ (9 months), Merkens et al.^47^ (15 cumulative years of recordings).

To our knowledge, this is the first time that a Mediterranean sperm whale study involving passive acoustic monitoring has been carried out covering such a long time period, across different seasons/years and in stereophony.

Figure 1 shows there is no seasonal cycle for the presence of sperm whales in this area. This species is present globally all year round. The months of February (2017–2018) are quite poor in terms of presence. Laran et al.^43^ had already analyzed on a monthly basis, the relative abundance of sperm whales in the Ligurian Sea, revealing year-round occurrences, peaks during the months of September and October, and larger social groups during winter. In our study, consistently with the latter, the densest observation of sperm whales (up to 9 individuals per day), occurred during the months of December 2016 to January 2017. The differences in attendance in the area between December 2016, 2017, and 2018 could be explained by variations in the Liguro-Provençal current. This current is stronger in winter (> 0.8 m s$^{-1}$), and weaker in summer (<0.5 m s$^{-1}$)^48^. When the current is strong, it might generate meanders^49^, which can lead to localised accumulation of organic matter. Thus in winter, when the current is strong, the increase of organic matter could lure sperm whales through its repercussions via the rest of the trophic chain.

On a daily basis, more sperm whales were detected at noon and fewer at 9 PM (Fig. 1). An estimation of the presence of sperm whales in a similar area has been assessed by André et al.^50^, and the maximum of detections was during the daylight hours. It could be possible that sperm whales move closer to the ridge slope areas (therefore within the sonobuoy detection range) during the day for foraging purposes. Indeed, several studies showed sperm whales have a preference for areas characterized by a particular seafloor topography (canyon and sea mouth) during the day^51–54^.

On the other hand, the measured daily pattern of noise levels shows a 3 dB increase of the noise around 3 AM and 9 PM, synchronous with the passages of ferries joining Corsica to the continent. This confirms the previous studies about the high level of anthropogenic noise in the Mediterranean Sea^55^, particularly near the coast^56^. Figure 3 shows a clear inverse pattern between the noise levels and sperm whale presence, consistently with other studies concerning the impact of ferries on cetacean species^50,57,58^. We suggest that these animals might purposely come to hunt in this area at times when no ferries are nearby, in order to avoid acoustic masking and increase their echolocation range.

This study allowed us to estimate the average density of sperm whales in the area: 1.69 whales/1000 km$^2$. Several studies have already estimated the abundance of sperm whales via acoustics: in the Tongue of The Ocean, Bahamas, the average density was 0.16 whales/1000 km$^2$^45^, 0.616 whales/1000 km$^2$ in the Northern Gulf of Mexico^59^, 1.44 whales/1000 km$^2$, in the Faroe Shetland Channel^60^, and between 1.26 and 4.25 whales/1000 km$^2$ in the Northeastern temperate Pacific^15^. Our density estimation in this geographical area is therefore consistent with the current bibliography on sperm whales. The relatively high measured density can be explained by the particular topography (presumably prone to feeding^51^) on which the buoy was installed.

Concerning the group size, in the current literature, the biggest group (called social unit) varies between 7^41^ and 15 individuals^52^. We observed a maximum of 9 tracks in a single passage, with the inconvenience that our current method cannot assert if they belong to the same social group or not, and whether two successive tracks come from the same individual. Regarding the IPI inferred sizes, the most observed category was from 9 to 12 m, represented by females and young males^32^. Previous works have already studied the different sizes of sperm whales in the Mediterranean Sea, using photo identification or IPI^31,32,61,62^. In the Atlantic Ocean, the estimated sizes of sperm whales ranged between 7 and 22 m^30^ (with 41 clicks), in New Zealand, the estimation was between 7 and 16 m^63^. In the East of the Mediterranean sea (the Ionian Sea), the sizes of sperm whales, are between 7.5 and 14 meters with a strong amount of animals ranging between 9 and 12 m (female or juvenile male)^32^. Our study, consistently with the latter, shows this population is mostly represented by adult females and immatures (55% for passage with one individual and 66.7% with 2 individuals). The largest recorded males have a size between 15 m and 16 m.

The main results of this study are summarized in Table 1, associated with the actual bibliography of this population.

**Table 1:** Main results of our study associated with the bibliography on the sperm whale population.

Topics	This study	Bibliography
Acoustic presence (AP) Counting individuals	422 sperm whales detected No seasonal cycle, sperm whales present during daytime Density of 1.69 whales/1,000 km$^2$ From 1 to 9 individuals	^31,41,43,50^
Background noise (BN)	Ambient noise louder at night on all octaves Season do not influence the ambient noise BN stronger during the periods corresponding to ferry crossings	^50,56^
BN and AP	Presence of sperm whales when ambient noise is low	^64^
Animal size/ IPI	More than half of individuals alone are adult females or juvenile males 8% of individuals alone are juveniles No seasonal / daily / lunar cycle on the sizes of individuals Juvenile passages are shorter in time (avg 71 min) than adult females/male (156 min) Single individuals are larger than individuals in a group	^32,34,61^.

Understanding the species distribution and its relation with anthropogenic noise will allow new management measures to be implemented on the coasts. Our results confirm the year-round presence of sperm whales, and thus the importance of the area. Further studies such as visual surveys could confirm the level of residency of the local population, and thus their dependence on the area. Moreover, new monitoring programs are being developed, such as a whale-ship collision mitigation system using a coastal network of buoys^65,66^.

With the presumed avoidance of whales from ferries, concrete measures could be considered and are urgently needed to reduce anthropic pressure, such as reduced ferry speeds or shifting of the ferry routes offshore to avoid areas of underwater canyons that are of importance for sperm whales^67^.

## Material and acoustic data acquisition

BOMBYX is a sonobuoy designed and installed in the Mediterranean sea^68^, near the island of Porquerolles (42$^\circ$56 N and 6$^\circ$19 E), in the South-East of France (Fig. 5). The position of the buoy is strategic, since it is part of both the Pelagos Sanctuary (the Sanctuary for Mediterranean Marine Mammals) and the french marine national park (Port-Cros). The Pelagos sanctuary is a protected marine area of 87,500 km$^2$, subject to an agreement between three countries (Italy, France, Monaco) for the protection of marines mammals^69^. This Sanctuary includes the coastal waters and pelagic area comprised between the headlands of the Giens peninsula to the Fosso Chiarone in southern Tuscany.

Numerous submarine canyons and seamounts are present in the Mediterranean Sea^70,71^, home to a great marine biodiversity^51,72,73^. In fact, upwelling currents follow this kind of bathymetry and cause the development of the entire food chain (plankton, fishes, mesopelagic squid and sperm whales)^74–76^. In Millot et al.^77^ the effect of the Mistral wind on the Ligurian current has been studied, showing that a frontal zone separates the Ligurian current and colder water upwelled from the Gulf of Lions. When the wind drops, the frontal zone moves Westward at higher speeds.

Thus, this area is frequented by several species of cetaceans (odontocetes and mysticetes), such as fin whales (*Balaenoptera physalus*), long-finned pilot whale (*Globicephala melas*), or the bottlenose dolphin (*Tursiops truncatus*). The most commonly observed species are the striped dolphin and the fin whale (as reported by aerial surveys)^78^ and according to the study of Drouot-Dulau et al. 2007, the sperm whales^79^ have been observed on this coast (between Monaco and Marseille).

The sonobuoy was therefore placed at the top of a vertical drop-off of 1500m depth. It is positioned at 25 m of depth and records at 50 kHz with two hydrophones spaced by 1.83 m. BOMBYX is facing south, meaning that the evolution of the TDoAs enables us to know if a group of sperm whales goes from east to west or west to east. The orientation of the buoy is relatively stable and its axis takes the direction of 230$^{\circ }$^80^. Since BOMBYX is fully immersed at 25 m of depth under the thermocline, the impact of surface noises is reduced. Its anchor is on the end of a terminal ridge of the continental slope to maximize the observations of offshore acoustic events.

A custom made sound card (by OSEAN SARL^68^) was used. The channel 1 hydrophone (east) is a Neptune D140 (up to 160 kHz) and the channel 2 hydrophone (west) is a D140, or a HTI (up to 80 kHz), depending on the sessions, with respectively − 207 ±2 versus − 206 ± 4 dB  re 1V/Pa @ 1 m. The recording protocol has changed over the years (varying between continuous recording to 5 min of recording every 20 min (meaning 15 min of pause), and between 24 and 16 bits encoding). Recording sessions lasted up to 3 months (Tab. S1 in Supplementary Material). Divers were regularly sent to change the batteries and collect the recordings. The first session started in May 2015 and the last one ended in December 2018.

## Method

**Figure 6:**
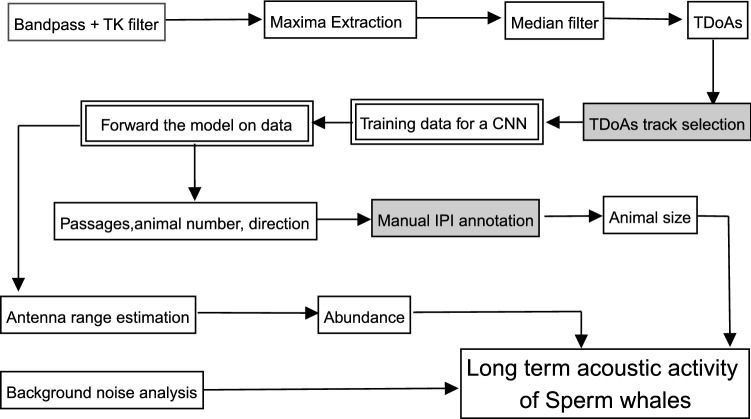
Summary diagram of the analysis. Gray boxes: semi-manual process. Double line: deep learning process.

The amount of data produced through the recording process is too large for an exhaustive human listening, and automatic detectors are not yet reliable enough to base behavioral statistics upon. Therefore, we set up an automatic sperm whale click detector joint with manual validation on the TDoAs tracks, then built an annotation system to extract the IPI from these clicks. The whole process of the methodology is described in Fig. 6, and can be done for any underwater stereo recording. The various tools used in this article are given in Python codes via online repositories.

### Click detector and manual annotation

To efficiently browse through the large number of recordings, we developed a custom-made annotation interface^65^. This interface first relies on a high recall but low precision click detector. By applying a Teager–Kaiser energy operator on the signal after a bandpass filter centered at 12.5 kHz^82^, most sperm whale clicks are detected, among other acoustic impulses such as pilot whale’s clicks, engine sounds, and others. We then computed the TDoAs of those detected impulses between the two hydrophones. An example of a sperm whale track in TDoAs is presented in Fig. S1 (Supplementary Material). The scatter plot of TDoAs over time allows the identification of localized acoustic emissions as clusters of points. Such a display allows the annotator to analyze 10 h of signal in one look, easily identifying any potential moving or stationary acoustic emitter.

To distinguish between sperm whales and other localized acoustic sources (boats or other cetaceans species), our interface allows us to observe TDoAs, select a detected impulse, plot the spectrogram of its surrounding signal as well as listen to it. This interface enabled the construction of a dataset consisting of 2313 sperm whale samples, 154 other cetaceans species samples, and 3087 noise samples, each of which belongs to individual files.

### Convolutional neural network for sperm whale detection

The data collected and annotated during the first part served to train a convolutional neural network (CNN) for sperm whale detection^65^. CNNs can be trained to learn the optimal parameters to classify data with a high degree of accuracy. CNNs use several layers of filters (or kernels) to convolve on the data sequentially, until a single confidence value is given. The model is trained to get the best fit of this confidence value with the given labels for each acoustic sample. In practice, training means optimizing the kernel weights with gradient descent iteratively. In this way, we thus optimize filters to discriminate between sample classes (here sperm whale versus any other sound), taking into account the large variety of noises and sperm whale clicks that are found in the dataset.

We designed a low complexity network (approximately 10 thousand parameters) that takes the Log Mel-Spectrum as an input, and consists of 3 depth-wise convolution layers^83^ of 128 kernels of size 7. The model was trained as a binary classifier (using a binary cross-entropy loss), a positive output identifying the presence of a sperm whale in the input recording. The dataset used for training contains annotations from 2015, 2016, and 2018 (932 clicks, 2091 boat noise, and 114 pilot whales/dolphins), and the test set contains the annotations of 2017 (with 996 examples of boats, 1331 examples of sperm whales, and 40 examples of pilot whales). To overcome the imbalance in training samples, sperm whale and other cetacean samples were weighted by 3 and 10 respectively (the weights are applied in the binary cross-entropy). The model showed a performance of 0.99 of area under the curve (AUC) on the training set and 0.94 of AUC on the test set. The Receiving Operator Curve (ROC) is shown in Supplementary Material Fig. S2.

Eventually, the trained model was forwarded over the whole dataset. The days featuring more than 40 CNN high confidence values (above 0.95) were manually validated. This process yielded 57 days with sperm whales (that the human annotator had missed due to noisy conditions), and 25 false positives (including 15 false positives coming from an especially noisy session due to an electronic malfunction). This combined process of manual annotation and click detection using machine learning yielded the sperm whale occurrence data shown in Fig. 1 and used in all statistics.

For the post-analysis, we defined sperm whales passages as periods when sperm whale clicks were heard in recordings in the annotation tool described in part “Click detector and manual annotation”. Sperm whale clicks were counted as separate passages when disjoint for at least 1 h.

### Inter-pulse interval (IPI) estimation and size measurement

#### Manual IPI annotation

The manual IPI annotation was done using another custom interface. It offers four complementary representations: the signal, the spectrogram, the autocorrelation, and the cepstrum. The user can use these 4 representations and listen to the part of the recording to check if it is sperm whale clicks. The annotations were confirmed by 3 annotators to reduce user bias. Each passage was annotated by at least 3 different experts and we have averaged the IPIs from the same track. It was not possible to calculate the IPI when there were more than 2 individuals in the passage.

#### From the IPI to a size measurement

The clicks emitted by sperm whales are made of multiple pulses. This particular click structure is explained by the bent horn model^23,24^, which describes the bouncing of the main pulse between two acoustic mirrors inside the sperm whale’s head. The IPI is the interval between two successive pulses or bounces, and its value is stable for each animal at a given time, as it is caused by the distance between the two acoustic mirrors^32,84^. Thus, the IPI can be used to estimate the size of a sperm whale^26,28,33^. A previous study^26^, suggested a relation between the Animal Size (AS) of the sperm whale and the IPI using the photogrammetry method:

1 $$\begin{aligned} AS = 4.833 + 1.453 \times IPI - 0.001 \times IPI^{2}. \end{aligned}$$

This Eq. (1) was built with 11 juveniles (less than 12 m) from the Azores and Sri Lanka. The latter is therefore effective for animals smaller than 11 m^85^. In 2011, a study proposed a new formula (2) to estimate the size of sperm whales over 11 m^28^:

2 $$\begin{aligned} AS = 1.258 \times IPI + 5.736. \end{aligned}$$

In this study, we test the two formulas. The sperm whale size gives us an insight about its sex and/or its sexual maturity^86^, grouped into three classes, immature male or female: *AS* < 9 m; adult female or juvenile male: 9 m < *AS* < 12 m; adult male: *AS* > 12 m.

IPI were extracted from all passages containing 1 or 2 individuals. To estimate the size of sperm whales, we applied Gordon’s equations^26^ and Growcott’s equations^28^ and we compared them. Since the size does not follow a normal distribution, the gaps between the equations were measured thanks to the Wilcoxon-Mann-Whitney test and showed a significant difference (*p value* = 0.023, Z = 8.89)^32^. For greater reliability of the results (the same method used in Caruso et al.^32^), the Gordon equation was used for the measurement of the size of animals with an IPI inferior to 4 ms, and the second equation (Growcott) for the sperm whales with an IPI superior to 4 ms. Figure S3 (Supplementary Material) shows the size distribution according to the 2 formulas for all annotated clicks.

### Background noise analysis

The noise level analysis was done on nine octaves bands, ranging from 50 to 12,800 Hz. For each octave, a sound pressure level (SPL) value was computed per recording. Only the East channel was analyzed since the west channel contains corrupt signals on some sessions. The full acquisition chain has been calibrated using Wenz curves^36^ to fit the standard noise level of the observed sea state. This was done in four steps. Firstly, the wave height *h* (in *m*) was computed from the wind speed *v*^87^ (in km h$^{-1}$) following Eq. (3) with *g* the standard acceleration due to gravity^88^.

3 $$\begin{aligned} h = v\frac{0.27}{g}. \end{aligned}$$

Secondly, each wave height (*h*) was converted to a sea state. Instead of using the quantified sea state, we chose to use a continuous version by fitting a curve on the sea state borders. Thirdly, the sea states were converted to noise level as Wenz curves^36^. A fitted version of those curves was also used due to the continuous sea states. Eventually, the noise level distribution (obtained via Wenz curves for the given sea state) was compared with the distribution of measured noise level in order to obtain the gain of the recording device for each session.

### Antenna range estimation and sperm whales average density

To estimate the sperm whales average density, an estimation of the antenna range is needed. The antenna range was deduced from the effective area of detection^89^ $a_e$, which is the product of *a*, the total area, and *p*, the probability of detecting an animal (Eq. 4).

4 $$\begin{aligned} a_e = a p. \end{aligned}$$

In our case, we estimated the detection probability (*p*) depending on the signal to noise ratio (SNR) given by Eq. (5), where *SL* is the source level, *G* is the directivity gain of the source, *NL* is the noise level, and *TL* is the transmission loss.

5 $$\begin{aligned} SNR = SL + G - TL - NL. \end{aligned}$$

For a given range *r* (distance between the sperm whale and the antenna), *TL* is given by Eq. (6), where $\alpha (f)$ is the acoustic water absorption at the frequency *f*.

6 $$\begin{aligned} TL = 20\log _{10}(r) + \alpha (f) r. \end{aligned}$$

A value of 1.43 dB km$^{-1}$ was used for the absorption, corresponding to a frequency of 12.5 kHz, a depth of 500 m, a temperature of 11 $^\circ \hbox {C}$, a salinity of 38.5 ppt and a pH of 8^90,91^.

For the directivity gain of the source *G* and the source level *SL*, the beam pattern described by Zimmer^92^ and Nosal^93^ were considered. Since the beam pattern described in Nosal has a relative amplitude, we added 161 dB to it to match the distribution described in Zimmer. The results presented in this paper only used the Zimmer beam pattern, as the Nosal beam pattern gives equivalent values.

**Figure 7:**
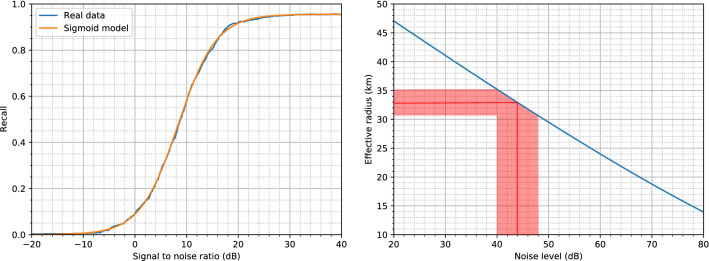
Left: recall of the CNN and the sigmoid model of this recall curve. Right: effective radius of the antenna for varying background noise level. The red line (zone) corresponds to the average (standard deviation) noise level at 12.8 kHz in the area.

The final component needed to evaluate the effective area is the recall of the CNN model (Fig. 7). The recall was obtained by doing multiple predictions on clicks with the addition of background noises sampled near them. For each click, the corresponding sampled background noise was added with multiple gains to obtain different SNR. To estimate the click and noise sound pressure level, we computed the root mean square (RMS) of the signal after a bandpass filter between 6 and 15 kHz. A time window of 2 ms centered on the main pulse of the click was used for the click, whilst the whole sampled noise signal was used for the noise level. Note that the unfiltered version of the signals was given to the neural network. Once the recall curve was obtained, a sigmoid curve was fitted onto it, in order to have a filter and continuous model for further computations.

To obtain the effective area $a_e$, Monte-Carlo simulations were used^94^. Each simulation was done by simulating $\frac{\pi }{4}2^{30}$ emissions spread uniformly in a 400 km radius. The maximum depth of the sperm whale was 1600 m, and the orientation was uniformly sampled in all directions^92^. Two depth distributions were tested. A uniform distribution and a log-normal distribution with a mean of 2.55 (354 m) and a standard deviation of 0.3. Both models gave similar results, thus only the uniform model is presented here. For each emission, (5) along with (6) were used to compute the SNR at the antenna depending on the parameters of the corresponding emissions. Thus, using the associated recall (probability of detecting the click), each simulation gave the expected value of the number of clicks received by the antenna. This divided by the number of clicks emitted in a simulation is the probability of detection of (4), which can be converted to an effective radius.

To estimate the population density, we used a methodology previously used for sperm whales and beaked whales^89^, formulated as Eq. (7) with *D* density, *n* average number of animals in a given area of size *a*.

7 $$\begin{aligned} D= \frac{n}{a}. \end{aligned}$$

### Statistical analysis

Various parameters were statistically tested to validate or invalidate the following correlations:

- Acoustics presence of sperm whales according to hours, to months, to the presence of ferry (Kruskall–Wallis test and Dunn–Bonferroni test)
- Sound pressure levels according to the months of the year, the seasons and the hours of the day (Kruskall–Wallis test and Dunn–Bonferroni test)
- Sound pressure level according to the presence/absence of sperm whale (Mann–Whitney test)
- Animal sizes according to the size of the group (Mann–Whitney test)
- Animal sizes according to the time, the sun and the moon phase (Mann–Whitney test)

The first test that was performed is the Shapiro-wilk to evaluate the normality of our distribution. The test rejects the hypothesis of normality when the p value is less than or equal to 0.05 (*p value* = 0.032, for periods of the day).

Our data were not normally distributed, so non-parametric tests were used to compare our samples: the Kruskall-Wallis test and the Wilcoxon–Mann–Whitney test have been used. The Wilcoxon–Mann–Whitney test is a non-parametric statistical test that tests the hypothesis that the medians of each of two groups of data are close and the Kruskall–Wallis test is used to determine if there are statistically significant differences between two or more groups of an independent variable on a continuous or ordinal dependent variable (non-parametric ANOVA).

If the p value of the Kruskall–Wallis Test is $\leqslant \alpha$: the differences between some of the medians are statistically significant, Post-hoc testing was used to evaluate differences between each distribution (Dunn–Bonferroni tests).

In this study, we compared the evolution of the sound pressure level according to the presence/absence of sperm whales (see Part “Sperm whale acoustic detection and background noise”). For this, we randomly selected 500 files with and without sperm whales to compare the distribution of decibels on all octaves^95^.

## Supplementary Information

Supplementary Information 1.

Supplementary Information 2.

Supplementary Information 3.
