# Supplementary material for: Passive acoustic monitoring of sperm whales and anthropogenic noise using stereophonic recordings in the Mediterranean Sea, North West Pelagos Sanctuary
Source: Sci Rep. 2022 Feb 7;12:2007. doi: 10.1038/s41598-022-05917-1 (PMC8821711; doi:10.1038/s41598-022-05917-1)
Supplement: Supplementary file 3 — Supplementary Information 3. [file 41598_2022_5917_MOESM3_ESM.pdf]

Supplementary material

Sessions	Start (dd/mm)	End (dd/mm)	Duration rec (min)	Duration pause (min)	Sampling (%)	Total duration (hh/min)
A	30/05/2015	31/07/2015	10	20	33	334h50
B	01/08/2015	25/10/2015	10	20	33	135h28
C	07/07/2016	05/08/2016	5	25	16	316h
D	17/11/2016	04/03/2017	1	5	16	340h
E	04/08/2017	16/10/2017	5	15	25	436h
F	21/12/2017	10/04/2018	5	15	25	661h
G	08/06/2018	11/11/2018	5	15	25	626h50
H	28/11/2018	26/12/2018	14	0	100	682h
Total						**3532h08**

Table S1. Summary of the records of all of the sessions. Total of around 147 days spread over 4 years (3532h).

Annotations tools

For this study, we have developed two semi-automatic annotation tools. The first was to display TDoAs tracks and to annotate the presence of sperm whale tracks (with a possibility of listening). The additional information of this interface (GUI, Readme, and Python code) is available at https://gitlab.lis-lab.fr/paul.best/tdoa_annot_bombyx.
The second annotation tool was designed for the IPI extraction (GUI, Readme, and Python code in the GIT repository: https://gitlab.lis-lab.fr/maxence.ferrari/ipi_annot. The Python code of this IPI annotator is split into two scripts. The first one, *ipi_extract.py*, is the core code to annotate any file. The second, *IPI_bombyx.py*, uses *ipi_extract.py* as a back-end and is made to facilitate access to the BOMBYX database, and the round-robin spread of the annotations to each annotator. It also displays a "next file" button, and shows online statistics.

The interface shows 20 s audio segment. Clicking on it will select the click that will be analyzed. The click selected will be at the maximum near the location clicked (0.1 s). The click will then be displayed in one of the 3 groups of plots selected by the radio button below them. Once assigned to a group, a click will be shown with 4 representations. The signal and spectrogram plots are linked and share the same P1 and IPI labels. The autocorrelation and cepstrum each allow an independent annotation of the IPI. An automatic IPI is also set at the local maxima in a range of 0.15 ms of the manual annotation. A vertical bar will follow the cursor while hovering above one of the plots. On the other plots of the same group, vertical bars are also displayed at the corresponding current hovering value. This allows the user to see if the IPI value that is going to be selected makes sense according to the other representations. A named label can also be assigned to a click.

Thus, for each click a maximum of 7 labels can be annotated: signal/spectrogram P1, signal/spectrogram IPI, manual autocorrelation IPI, automatic autocorrelation IPI, manual cepstrum IPI, automatic cepstrum IPI, and individual label.

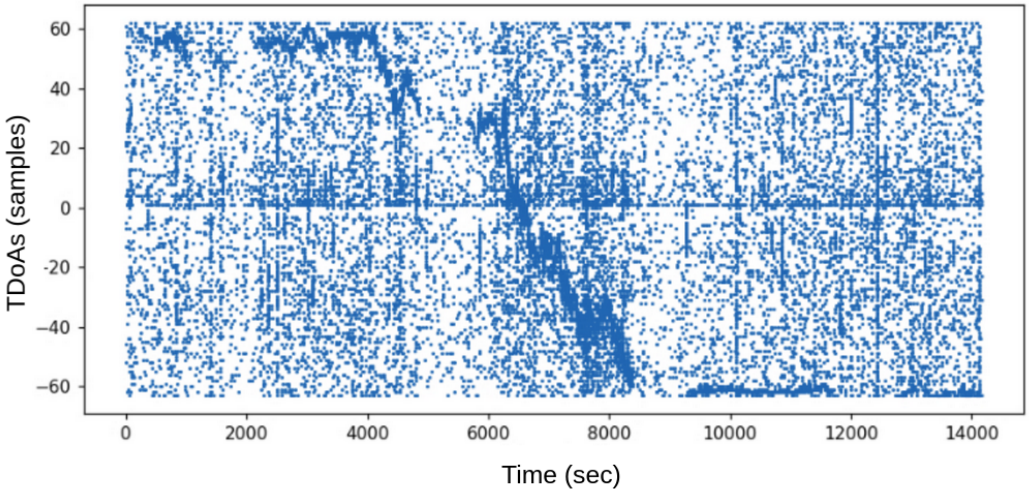

Figure S1. Example of a TDoAs track of one individual.

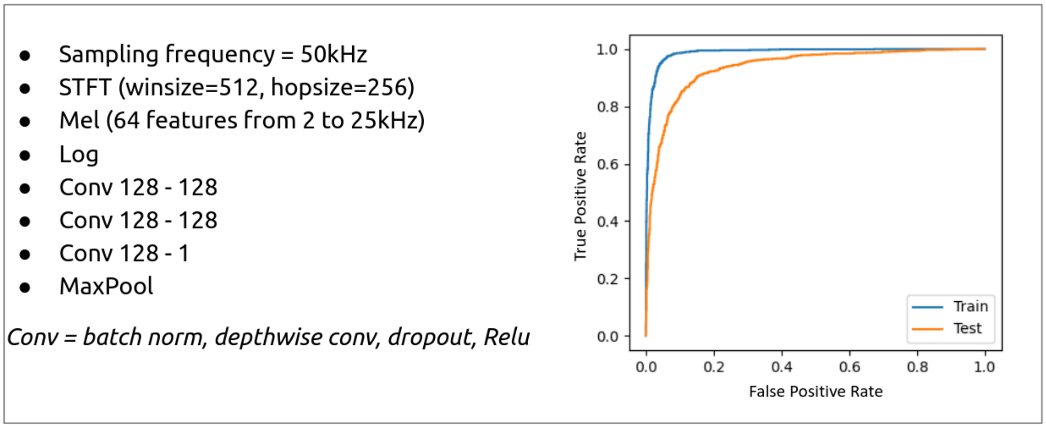

Figure S2. Model's architecture (left), and Receiving Operator Curves for the model (right).

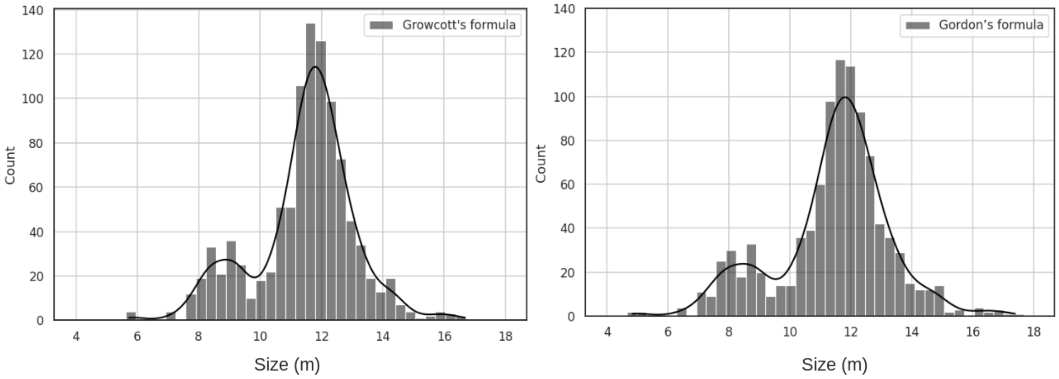

Figure S3. The size of the sperm whales for each IPI (from passages with one individual) according to Growcott's formula (left), and Gordon's formula (right).

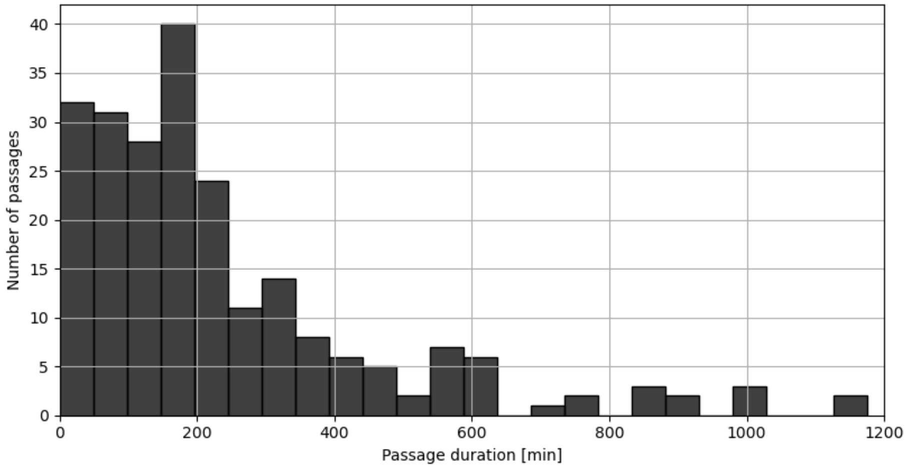

Figure S4. Distribution of the passages duration.

Two recordings samples are available in "Supplementary Dataset" files (16 bits SR: 50kHz). In the first, only one individual is present, and in the second, two sperm whales emit clicks.
